# A History of Electrotherapy
*An Essay on the History of Electrotherapy and Diagnosis. By Hector A. Colwell, M.B. Pp. 180. Illustrated (Heinemann, 17s. 6d. net.)


**Published:** 1922-04

**Authors:** 


### THE REVIEW OF THE MONTH.

### A HISTORY OF ELECTROTHERAPY.*

WHEN we see our streets and houses lighted by electricity, our railway trains and tramcars run by the same power, and huge manufactories deriving their supply of energy from the same source, it is difficult to realise that all these undertakings have been developed from the simple experiments and apparatus of the past, which, a few years ago, were little more than scientific playthings. The history of the genesis of this knowledge is full of interest. Electricity did not spring, like Athene of old, in the full panoply of power from the brain of one supreme genius, but each of many workers has added his little fragment to make the sum of the knowledge we now possess. Never was there a better illustration of the truth of the saying that "knowledge is power." It redounds to the honour of the medical profession that so many of the experimenters have be n members of it, who, though groping in the dark, were earnest in their endeavours to apply electricity to the relief of human suffering. The story of this evolution of the science was therefore well worth the telling, and has found a sympathetic historian in Dr. Colwell. The very word "electric" we owe to Dr. William Gilbert, of Colchester, President of the Royal College of Physicians, and Physician to Queen Elizabeth, who exercising her Royal prerogative, granted him a pension. It appears that the noun "Electricity" is due to two other English observers—viz., Charleton and Robert Boyle, whose name has been immortalised in the well-known "Boyle's Law." But the fraternity of medical investigators is cosmopolitan. A French physician, Du Fay, was the first to draw sparks from an electrified human body—his own—suspended to the ceiling by cords of silk. Dr. Jallabert, of Geneva (*"Le père de l'électricité médicale"*), first applied electricity to produce contraction in the muscles of a paralysed limb (1747). Yet centuries before, the natural electricity of the Ray skate appears to have been used as a remedy for disease, as is testified by Pliny, Paulus Aegineta and Dioscorides. It will probably be news to many of us that John Wesley published one of the earliest books on electro-therapeutics in the English language, and that the revolutionary Jean Paul Marat, when practising as a physician in England, was a pioneer in this direction. The London hospitals were early in the field, and in 1767 the Middlesex Hospital purchased an electrical apparatus, an example which was followed ten years later by St. Bartholomew's. But quackery, too, was not far behind, and James Graham, with his "Celestial bed," and Elisha Perkins with his "Metallic tractors," were precursors of a whole tribe of charlatans, whose electropathic belts and other worthless paraphernalia, still advertised in the columns of many local newspapers, bear eloquent witness to the magnitude of human credulity.

It was not, however, until Faraday discovered electro-magnetic induction that real scientific progress was made, and the modern types of coils and batteries were invented. The electric cautery was introduced shortly before 1850, in which year it was first used in England by Mr. Marshall, at University College Hospital. Space fails us to recount even the bare names of those who have added their quota to our present knowledge since then. Althaus, Duchenne, Benjamin Ward Richardson, Leduc, Steavenson, Lewis Jones, Röntgen, Becquerel, Curie and the rest—truly a galaxy of great minds. For the contributions of each and of others we must refer the reader to Dr. Colwell's book, the moral of which is to be found in his concluding sentence: "The great need at the present time in this country is the establishment of a central institute where such researches can be conducted by qualified workers, where apparatus can be standardised, and adequate means of protection devised to meet the ever increasing power at our disposal." In tracing the growth of the science of electro-therapy and diagnosis the words of the Preacher rise to our minds with peculiar insistence and force: "There be of them that have left a name behind them, that their praises might be reported. And some there be, which have no memorial; who are perished, as though they had never been." Dr. Colwell's book will be found of great interest.—G. A.

*An Essay on the History of Electrotherapy and Diagnosis. By Hector A. Colwell, M.B. Pp. 180. Illustrated. (Heinemann, 17s. 6d. net.)

